# Direction of Arrival Estimation for MIMO Radar via Unitary Nuclear Norm Minimization

**DOI:** 10.3390/s17040939

**Published:** 2017-04-24

**Authors:** Xianpeng Wang, Mengxing Huang, Xiaoqin Wu, Guoan Bi

**Affiliations:** 1 State Key Laboratory of Marine Resource Utilization in South China Sea, Hainan University, Haikou 570228, China; wxpeng1986@126.com (X.W.); xq-wu@hainu.edu.cn (X.W.); 2 College of Information Science and Technology, Hainan University, Haikou 570228, China; 3 School of Electrical and Electronic Engineering, Nanyang Technological University, Singapore 639798, Singapore; egbi@ntu.edu.sg

**Keywords:** multiple-input multiple-output radar, noncircular signal, direction of arrival estimation, nuclear norm minimization, unitary transformation

## Abstract

In this paper, we consider the direction of arrival (DOA) estimation issue of noncircular (NC) source in multiple-input multiple-output (MIMO) radar and propose a novel unitary nuclear norm minimization (UNNM) algorithm. In the proposed method, the noncircular properties of signals are used to double the virtual array aperture, and the real-valued data are obtained by utilizing unitary transformation. Then a real-valued block sparse model is established based on a novel over-complete dictionary, and a UNNM algorithm is formulated for recovering the block-sparse matrix. In addition, the real-valued NC-MUSIC spectrum is used to design a weight matrix for reweighting the nuclear norm minimization to achieve the enhanced sparsity of solutions. Finally, the DOA is estimated by searching the non-zero blocks of the recovered matrix. Because of using the noncircular properties of signals to extend the virtual array aperture and an additional real structure to suppress the noise, the proposed method provides better performance compared with the conventional sparse recovery based algorithms. Furthermore, the proposed method can handle the case of underdetermined DOA estimation. Simulation results show the effectiveness and advantages of the proposed method.

## 1. Introduction

Recently, the parameter estimation problem has attracted more and more attentions in multiple input multiple output (MIMO) radar [1,2,3,4], especially for DOA estimation. Compared with the conventional phased-array radar systems, MIMO radar exhibits better parameter estimation performance by using the spatial diversity gain and/or waveform diversity gain. In general, there are two classes of MIMO radar: statistical MIMO radar [5] and colocated MIMO radar [6]. The statistical MIMO radar is equipped with separated antennas in both transmit and receive arrays, which can achieve the spatial diversity gain from different channels. The transmit and receive antennas of the colocated MIMO radar are closely located to form a virtual array with a large aperture and achieve higher spatial resolution. In this paper, we consider the DOA estimation problem in colocated MIMO radar.

For the DOA estimation problem in MIMO radar, the algorithms can be mainly divided into two groups: subspace-based algorithms and sparse recovery (SR) based algorithms. In the subspace-based algorithms, the multiple signal classification (MUSIC) algorithm and its variations have been investigated [7,8]. In general, these algorithms can estimate the DOA via searching the spatial spectrum at heavy computational costs. On the other hand, the estimation of signal parameters via rotational invariance technique (ESPRIT) and its extensions are proposed in [9,10], which can achieve the DOA by calculating the rotation invariant factor. Compared with MUSIC algorithm, the ESPRIT algorithm requires lower computational complexity due to the avoidance of the extensive spatial searching, but it can only be applied to some special array configurations, such as uniform linear arrays (ULAs). Due to the fact that the subspace-based algorithms are implemented with the eigenvalue decomposition (EVD) of the covariance matrix, these algorithms generally need some necessary conditions, such as a reasonably large number of snapshots and high enough signal-to-noise ratio (SNR), to achieve the desirable performance. In recently years, the SR-based techniques are investigated in both conventional phase-array systems [11,12,13,14,15] and MIMO radar systems [16,17,18,19], which exploit the concept of sparse signal reconstruction. In [11], a sparse signal reconstruction perspective based on $l_{1}$-norm penalty is successfully proposed for DOA estimation, in which the computational complexity of the sparse reconstruction and the sensitivity of the measurement noise are reduced by using the singular value decomposition (SVD) technique. Some other sparsity-inducing techniques, such as covariance vectors based SR algorithm [12], covariance matrix based SR algorithm [13], real-valued SR algorithm [14] and coprime array based sparse representation algorithm [15], are investigated in phased-array systems. In addition, several SR-based algorithms are proposed for DOA estimation in MIMO radar [16,17,18,19]. The simulation results in [11,12,13,14,15,16,17,18,19] have shown that the SR-based DOA estimation methods provide better performance than the subspace-based algorithms in the cases using a limited number of snapshots and low SNR.

It is well known that the spatial resolution depends on the array aperture, i.e., larger array aperture means better performance. In radar systems, exploiting the property of complex noncircular signals, such as BPSK, ASK, and UQPSK modulated signals, provides a possible way to enlarge the virtual aperture for improving the performance, which has been verified in [20,21,22]. For MIMO radar, some subspace-based methods are extended for DOA estimation by using the noncircular property of signals, and the theoretical analysis and simulation results verify that these methods achieve higher spatial resolution and better performance than traditional subspace-based methods [23,24,25]. On the other hand, there are a few literatures about the SR-based DOA estimation by using the noncircular property of signals. In [26], a SR-based DOA estimation algorithm for noncircular sources is investigated by combining the received data and its conjugation, and the performance is improved significantly compared with the conventional SR-based methods. In [27], a nuclear norm minimization (NNM) framework is proposed to effectively use the whole aperture corresponding to the extended data. Therefore it provides better angle estimation performance than the SR-based method in [28].

In this paper, a novel unitary nuclear norm minimization (UNNM) algorithm is proposed for DOA estimation of noncircular sources in MIMO radar. The UNNM algorithm can be seen as a real-valued extension of the nuclear norm minimization (NNM) algorithm in [27]. In the proposed method, the virtual array aperture can be doubled by using the noncircular properties of signals, and the complex extended data can be turned into a real-valued data by utilizing unitary transformation. Then the real-valued data are represented with a block-sparse model based on a novel over-complete dictionary, and a UNNM algorithm is formulated for recovering the block-sparse matrix. In order to achieve the enhanced sparsity of solutions, the real-valued NC-MUSIC spectrum is exploited to design the weight matrix for reweighting the nuclear norm minimization. Finally, the DOA is achieved by using the recovered matrix. Due to the suppression of the noise by using the real-valued structure, the proposed method shows higher spatial resolution and better performance than NNM algorithm. In addition, the proposed method can handle the case of underdetermined DOA estimation because of using the whole extended aperture.

This paper is organized as follows. The data model and problem formation are introduced in Section 2. A unitary nuclear norm minimization algorithm is proposed in Section 3, and several related issues are discussed in Section 4. The simulation results are given and analyzed in Section 5. Section 6 gives the conclusion of the paper.

*Notation*: ${\left(\cdot \right)}^{H}$, ${\left(\cdot \right)}^{T}$, ${\left(\cdot \right)}^{-1}$, ${\left(\cdot \right)}^{*}$, ${\left(\cdot \right)}^{+}$ and Re{·} represent the conjugate-transpose, transpose, inverse, conjugate, pseudo-inverse and real part operator, respectively. ⊗ and ⊙ denote the Kronecker product and Khatri-Rao product, respectively. $I_{K}$ denotes a K×K dimensional unit matrix. diag{·} denotes the diagonal matrix, and blkdiag{A,B} represents a block diagonal matrix with diagonal entries A and B. det{A} is the determinant of the square matrix A, and $\left|\right|\cdot {\left|\right|}_{F}$ denotes the Frobenius norm.

## 2. Data Model and Problem Formulation

**Figure 1:**
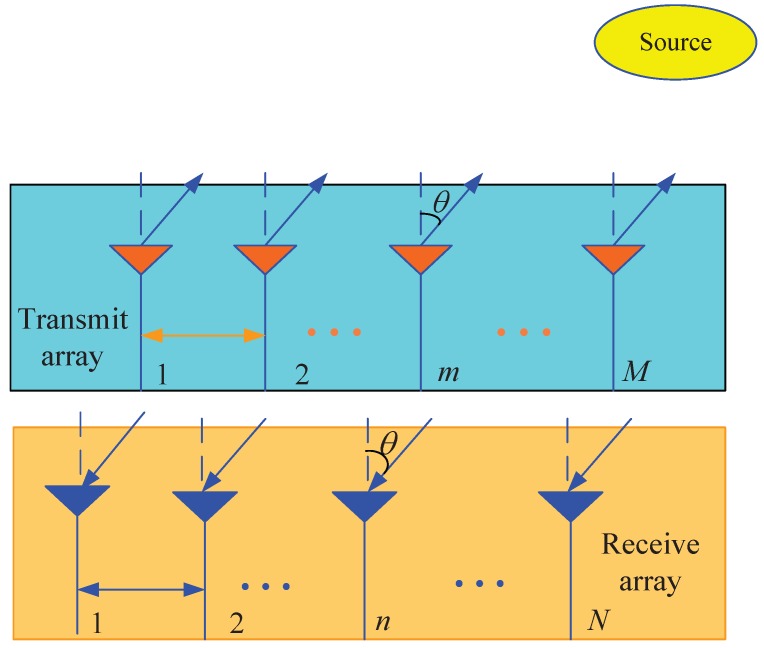
The configuration of colocated MIMO radar.

We consider a MIMO radar with *M* antennas in the transmit array and, *N* antennas in the receive array, and all the antennas are omnidirectional. The transmit and receive arrays are half-wavelength spaced uniform linear arrays (ULAs) and located closely, as shown in Figure 1. Thus, the DOAs of a source with respect to the normals of transmit and receive arrays are the same. The transmit array uses *M* antennas to emit *M* strictly noncircular waveforms, such as BPSK modulated signals, which are orthogonal and have identical bandwidth and central frequency. It is assumed that there are *P* sources in the far-field, and the DOA of *p*th source is denoted as $\theta_{p}$. To exploit the orthogonality of the noncircular waveforms, a group of matched filters can be formed. Then the received data of the receive array are handled with matched filters, and the output of all the matched filters can be expressed as [23,24,25,26,27].

(1) $$y\left(t\right)=As_{c}\left(t\right)+n\left(t\right)$$

where $y\left(t\right)\in C^{MN\times 1}$ is the received data vector. $A=A_{t}⊙A_{r}\in C^{MN\times P}$ is the transmit-receive steering matrix, $A_{t}=\left[a_{t}\left(\theta_{1}\right),\cdots ,a_{t}\left(\theta_{P}\right)\right]\in C^{M\times P}$ and $A_{r}=\left[a_{r}\left(\theta_{1}\right),\cdots ,a_{r}\left(\theta_{P}\right)\right]\in C^{N\times P}$ are the transmit and receive steering matrices, respectively. $a_{t}\left(\theta_{p}\right)={\left[1,\exp\left(j\pi \sin\theta_{p}\right),\cdots ,\exp\left(j\pi \left(M-1\right)\sin\theta_{p}\right)\right]}^{T}$ and $a_{r}\left(\theta_{p}\right)=[1,\exp\left(j\pi \sin\theta_{p}\right),\cdots ,$$\exp\left(j\pi \left(N-1\right)\sin\theta_{p}\right){]}^{T}$ are the transmit and receive steering vectors, respectively. $n\left(t\right)\in C^{MN\times 1}$ is the additional Gaussian white noise vector with the covariance matrix $\sigma^{2}I_{MN}$. $s_{c}\left(t\right)$ is strictly noncircular signal vector, and it is generally written as

(2) $$s_{c}\left(t\right)=\Phi s_{0}\left(t\right)$$

where $s_{0}\left(t\right)\in R^{P\times 1}$ is a real-valued symbol vector, and $\Phi =\mathrm{diag}\left\{\left[\exp\left(j\psi_{1}\right),\cdots ,\exp\left(j\psi_{P}\right)\right]\right\}\in C^{P\times P}$ is a diagonal matrix that contains the noncircularity phase $\psi =[\psi_{1},\cdots ,\psi_{P}]$, which can be arbitrary for each source. Then substituting Equation (2) into Equation (1), we have

(3) $$y\left(t\right)=A\Phi s_{0}\left(t\right)+n\left(t\right).$$

In the case of *L* snapshots, the received data in Equation (3) are rewritten as

(4) $$Y=A\Phi S_{0}+N$$

where $Y=\left[y\left(t_{1}\right),\cdots ,y\left(t_{L}\right)\right]\in C^{MN\times L}$ is the received data matrix, $S_{0}=\left[s_{0}\left(t_{1}\right),\cdots ,s_{0}\left(t_{L}\right)\right]$ is the real-valued symbol matrix, and $N=[n\left(t_{1}\right),\cdots ,n\left(t_{L}\right)]$ is the additional Gaussian white noise matrix.

## 3. Unitary Nuclear Norm Minimization Algorithm

In this section, the redundant elements of the virtual array in MIMO radar are firstly eliminated, and a real-valued extended data model is established by using the noncircular properties of the signals and unitary transformation. Then a block-sparse model is formulated to represent the real-valued extended data, and a reweighted nuclear norm minimization algorithm is proposed for DOA estimation.

### 3.1. Augmented Data Matrix and Unitary Transforation

Due to the configuration of MIMO radar shown in Figure 1, there are some redundant elements in virtual array. The reduced dimensional transformation technique can be adopted to eliminate these redundant elements, and the reduced dimension transformation matrix is given as [10]

(5) $$D={\left(G^{H}G\right)}^{-(1/2)}G^{H}$$

where the transformation matrix G is expressed as

(6) $$G={\left[J_{0}^{T},J_{1}^{T},\cdots ,J_{M-1}^{T}\right]}^{T}$$

(7) $$J_{m}=\left[0_{N\times m},I_{N},0_{N\times (M-m-1)}\right]\;m=0,1,...,M-1$$

and satisfies $a_{t}\left(\theta \right)⊗a_{r}\left(\theta \right)=\mathrm{Gb}\left(\theta \right)$, where b(θ)=[1,$\exp\left(j\pi \sin\theta_{p}\right),\cdots ,\exp\left(j\pi \left(M+N-2\right)\sin\theta_{p}\right){]}^{T}\in C^{(M+N-1)\times L}$ is the steering vector without the redundant elements. Multiplying D with the data matrix Y in Equation (4) yields

(8) $$\begin{array}{c} X=DY={\left(G^{H}G\right)}^{\left(1/2\right)}B\Phi S_{0}+DN\\ =F^{\left(1/2\right)}B\Phi S_{0}+\bar{N}=\bar{B}\Phi S_{0}+\bar{N} \end{array}$$

where $\bar{B}=F^{\left(1/2\right)}B$ is the new steering matrix composed with the steering matrix $B=[b\left(\theta_{1}\right),\cdots ,b\left(\theta_{P}\right)]$ and the weight matrix $F=G^{H}G=\mathrm{diag}\{[1,2,...,$ $\underset{|M-N|+1}{\underset{︸}{\min(M,N),...,\min(M,N)}},....,2,\;$1]}. $\bar{N}=DN$ is the Gaussian white noise matrix after using the reduced dimensional transformation technique. In order to take advantage of the noncircular properties of the signals, the common preprocessing step is applied to the data matrix X. Then the 2Q×L(Q=M+N−1) augmented matrix can be achieved as [23,24,25,26,27]

(9) $$\begin{array}{cc} Y_{E} & =\left[\begin{array}{c} \Pi_{Q}{\bar{B}}^{*}\Phi^{*}\\ \bar{B}\Phi \end{array}\right]S_{0}+\left[\begin{array}{c} \Pi_{Q}{\bar{N}}^{*}\\ \bar{N} \end{array}\right]=B_{E}S_{0}+N_{E} \end{array}$$

where $\Pi_{Q}$ is the Q×Q exchange matrix with ones on its anti-diagonal elements and zeros elsewhere, and $N_{E}$ is the 2Q×L augmented noise matrix. $B_{E}=\left[b_{e}\left(\theta_{1},\psi_{1}\right),\cdots ,b_{e}\left(\theta_{P},\psi_{P}\right)\right]\in C^{2Q\times P}$ is the augmented steering matrix with the extended steering vector $b_{e}\left(\theta_{p},\psi_{p}\right)=[{\left(\Pi_{Q}F^{\left(1/2\right)}b^{*}\left(\theta_{p}\right)\exp\left(-j\psi_{P}\right)\right)}^{T},$${\left(F^{\left(1/2\right)}b\left(\theta_{p}\right)\exp\left(j\psi_{P}\right)\right)}^{T}{]}^{T}$, which enlarges the virtual array aperture to achieve performance improvement. The NNM algorithm in [27] is implemented with complex-valued processing based on Equation (9). Because the complex multiplication generally using three or four real multiplications, a considerable amount of computational complexity can be reduced if the complex-valued problem in Equation (9) can be transformed into a real-valued one. In addition, it has been indicated in [14] that the noise can be suppressed by using the real-valued structure. These merits motivate us to propose a real-valued sparse recovery framework for DOA estimation.

Following the convention in [28], the unitary transformation matrix used in the following section can be defined as

(10) $$U_{2K}=\frac{1}{\sqrt{2}}\left[\begin{array}{cc} I_{K} & jI_{K}\\ \Pi_{K} & -j\Pi_{K} \end{array}\right]$$

and

(11) $$U_{2K+1}=\frac{1}{\sqrt{2}}\left[\begin{array}{ccc} I_{K} & 0 & jI_{K}\\ 0^{T} & \sqrt{2} & 0^{T}\\ \Pi_{K} & 0 & -j\Pi_{K} \end{array}\right].$$

It is obvious that the transformation matrix satisfies $U_{M}U_{M}^{H}=I_{M}$, $U_{M}=\Pi_{M}U_{M}^{*}$ and ${\left(\Pi_{M}U_{M}\right)}^{T}=U_{M}^{H}$. Then we have the following *Lemma 1* and *Lemma 2* .

**Lemma** **1.** For any complex matrix $K\in C^{M\times N}$, if $K=\Pi_{M}K^{*}$, $U_{M}^{H}K$ is real.

**Proof.**  The conjugation of $U_{M}K$ is expressed as

(12) $$\begin{array}{cc} {\left(U_{M}^{H}K\right)}^{*} & ={\left(U_{M}^{H}\Pi_{M}\Pi_{M}K\right)}^{*}\\ & ={\left(\Pi_{M}U_{M}\right)}^{T}{\left(\Pi_{M}K\right)}^{*}=U_{M}^{H}K. \end{array}$$

Thus, $U_{M}^{H}K$ is real, which results in Lemma 1. ☐

**Lemma** **2.** For any centro-Hermitian $K\in C^{M\times N}$, i.e., $K=\Pi_{M}K^{*}\Pi_{N}$, $U_{M}^{H}KU_{N}$ is real.

**Proof.**  The conjugation of $U_{M}^{H}KU_{N}$ is written as

(13) $$\begin{array}{cc} {\left(U_{M}^{H}KU_{N}\right)}^{*} & ={\left(U_{M}^{H}\Pi_{M}\Pi_{M}K\Pi_{N}\Pi_{N}U_{N}\right)}^{*}\\ & ={\left(\Pi_{M}U_{M}\right)}^{T}\left(\Pi_{M}K^{*}\Pi_{N}\right)\left(\Pi_{N}U_{N}^{*}\right)\\ & =U_{M}^{H}KU_{N}. \end{array}$$

So, $U_{M}^{H}KU_{N}$ is equal to its conjugation and is hence real, which results in Lemma 2. ☐

According to the Lemma 1, it is easy to check that the vector ${\bar{b}}_{e}\left(\theta_{p},\psi_{p}\right)=U_{2Q}b_{e}\left(\theta_{p},\psi_{p}\right)$ is real. Then the received data matrix can be constructed by using unitary transformation, which is shown as

(14) $$\begin{array}{cc} U_{2Q}Y_{E} & =U_{2Q}B_{E}S_{0}+U_{2Q}N_{E}\\ & ={\bar{B}}_{E}S_{0}+U_{2Q}N_{E} \end{array}$$

where ${\bar{B}}_{E}=U_{2Q}B_{E}$ is the real-valued extended steering matrix. According to Equation (14), the data $U_{2Q}Y_{E}$ can be divided into real and imaginary parts

(15) Re(U2QYE)=B¯ERe(S0)+Re(U2QNE),

(16) Im(U2QYE)=B¯EIm(S0)+Im(U2QNE).

Then combing Equations (15) and (16), a new real-valued extended data matrix is written as

(17) $$\begin{array}{c} Z_{r}={\bar{B}}_{E}{\bar{S}}_{0}+N_{r} \end{array}$$

where S¯0=[Re(S0)Im(S0)] and Nr=[Re(U2QNE)Im(U2QNE)]. Noting that the real-valued extended steering matrix ${\bar{B}}_{E}$ contains the unknown noncircularity phase and its conjugation, the complete dictionary can not be formulated for the sparse representation framework. In order to achieve an efficient dictionary without the influence of the unknown noncircularity phase, the augmented steering matrix can be rewritten as

(18) $$\begin{array}{c} B_{E}=A_{E}\Phi_{E} \end{array}$$

with

(19) $$\begin{array}{c} A_{E}=\left[A_{e}\left(\theta_{1}\right),\cdots ,A_{e}\left(\theta_{P}\right)\right],\; \end{array}$$

and

(20) $$\begin{array}{c} \Phi_{E}=\mathrm{blkdiag}\left\{\left[\phi_{1},\cdots ,\phi_{P}\right]\right\} \end{array}$$

where $\phi_{p}={\left[\exp\left(-j\psi_{p}\right),\exp\left(j\psi_{p}\right)\right]}^{T},\left(p=1,2,\cdots ,P\right)$, and $A_{e}\left(\theta_{p}\right)$ is expressed as

(21) $$\begin{array}{c} A_{e}\left(\theta_{p}\right)=\left[\begin{array}{cc} \Pi_{Q}F^{1/2}b^{*}\left(\theta_{p}\right) & 0_{(M+N-1)\times 1}\\ 0_{(M+N-1)\times 1} & F^{1/2}b\left(\theta_{p}\right) \end{array}\right]. \end{array}$$

According to Equation (21), the steering submatrix satisfies $A_{e}\left(\theta_{p}\right)=\Pi_{2Q}A_{e}^{*}\left(\theta_{p}\right)\Pi_{2}$. Following Lemma 2, the corresponding real-valued submatrix is expressed as

(22) $$\begin{array}{c} A_{r}\left(\theta_{p}\right)=U_{2Q}A_{e}\left(\theta_{p}\right)U_{2}. \end{array}$$

Then the real-valued extended steering matrix ${\bar{B}}_{E}$ can be rewritten as

(23) $$\begin{array}{c} {\bar{B}}_{E}=A_{r}\Phi_{r} \end{array}$$

where $A_{r}=\left[A_{r}\left(\theta_{1}\right),\cdots ,A_{r}\left(\theta_{P}\right)\right]$ and $\Phi_{r}=\mathrm{blkdiag}\left\{\left[{\bar{\phi }}_{1},\cdots ,{\bar{\phi }}_{P}\right]\right\}$ with real-valued vector ${\bar{\phi }}_{p}=U_{2}^{H}\phi_{p}$. Substituting Equation (23) into Equation (17) yields

(24) $$\begin{array}{c} Z_{r}=A_{r}\Phi_{r}{\bar{S}}_{0}+N_{r}=A_{r}S_{r}+N_{r} \end{array}$$

where $S_{r}=\Phi_{r}{\bar{S}}_{0}$ can be seen as a new real-valued signal matrix, and $A_{r}$ is the new real-valued steering matrix without the unknown noncircularity phase. Thus, the signal model in Equation (24) is suitable to formulate the sparse representation framework.

### 3.2. Nuclear Norm Minimization Algorithm

In order to reduce the data dimension and the sensitivity of the noise, the SVD technique is applied to the real-valued data. Let $V_{s}\in R^{2L\times P}$ be a matrix composed of the right singular vectors corresponding to the *P* largest singular values, then multiplying the real-valued data $Z_{r}$ by $V_{s}$ yields

(25) $$\begin{array}{c} Z_{rs}=Z_{r}V_{s}=A_{r}S_{r}V_{s}+N_{r}V_{s}=A_{r}S_{rs}+N_{rs} \end{array}$$

where $N_{rs}=N_{r}V_{s}$, and $S_{rs}=\Phi_{r}{\bar{S}}_{0}V_{s}=\Phi_{r}{\bar{S}}_{s0}={\left[S_{rs1}^{T},\cdots ,S_{rsP}^{T}\right]}^{T}$ is a block structure, which is divided into *P* rank one matrices $S_{rsp}={\bar{\phi }}_{p}{\bar{s}}_{sp}\;\left(p=1,2,\cdots ,P\right)$, where ${\bar{s}}_{sp}$ is the *p*th row of ${\bar{S}}_{s0}$. It should be highlighted that the real-valued steering matrix $A_{r}$ in Equation (25) does not contain any information of the unknown noncircularity phase. Then using the view of the sparse representation, Equation (25) can be represented with a overcomplete dictionary by discretizing the interested spatial domain. Let the discretized spatial sampling grid be ${\left\{{\bar{\theta }}_{l}\right\}}_{l=1}^{K}\left(K\gg P\right)$, then the real-valued overcomplete dictionary can be constructed as $A_{\bar{\theta }}=\left[A_{r}\left({\bar{\theta }}_{1}\right),\cdots ,A_{r}\left({\bar{\theta }}_{K}\right)\right]\in R^{2Q\times 2K}$. Based on the dictionary, the sparse representation model of the data in Equation (25) is expressed as

(26) $$\begin{array}{c} Z_{rs}=A_{\bar{\theta }}S_{\bar{\theta }}+N_{rs} \end{array}$$

where $S_{\bar{\theta }}=\left[S_{\bar{\theta }1},S_{\bar{\theta }2},\cdots ,S_{\bar{\theta }K}\right]$ is a block-sparse real-valued matrix, and *P* nonzero blocks in the matrix are equal to $S_{rsp}\;\left(p=1,2,\cdots ,P\right)$. The positions of these nonzero blocks are used to estimate the DOA. Thus, the DOA estimation issue is turned into the problem of recovering the block-sparse matrix. It is noticed that the block-sparse matrix $S_{\bar{\theta }}$ has two types of different sparsity: sparse block and sparse rank in each block (rank one or rank zero). In order to combine the two types of sparsity to recover the block-sparse matrix, a convex nuclear norm minimization problem is formulated as

(27) $$\begin{array}{c} \min_{S_{\bar{\theta }}}\sum_{l=1}^{K}\left|\right|S_{\bar{\theta }l}{\left|\right|}_{*}\\ s.t.\;||Z_{rs}-A_{\bar{\theta }}S_{\bar{\theta }}{\left|\right|}_{F}\leq \beta \end{array}$$

where β is regularization parameter and sets the amount of error. The nuclear norm in Equation (27) is defined by

(28) $$\begin{array}{c} \left|\right|S_{\bar{\theta }k}{\left|\right|}_{*}=\sum_{\ell =1}^{\min(2,P)}\zeta_{\ell }\left(S_{\bar{\theta }k}\right) \end{array}$$

where $\zeta_{\ell }\left({\tilde{S}}_{\psi_{k}}\right)$ denotes the *ℓ*th singular value of the block matrix $S_{\bar{\theta }k}$. The convex nuclear norm minimization problem can be solved by semidefinite programming [29]. According to Equation (27), the nuclear norm minimization problem can be seen as the $l_{1}$ norm penalty of $\left|\right|S_{\bar{\theta }k}{\left|\right|}_{*}\;\left(k=1,2,\cdots ,K\right)$. Thus, the sparsity of the solution is limited due to the inherent property of $l_{1}$ norm penalty, which causes the loss of DOA estimation performance. In order to solve this problem, a weight matrix based on a novel real-valued NC-MUSIC spectrum is formulated for reweighting the nuclear norm minimization, which can achieve the enhanced sparsity of solutions. Let $U_{n}\in R^{2Q\times P}$ be a matrix composed of the left singular vectors corresponding to 2Q−P smallest singular values, i.e., the noise subspace $E_{n}$. In addition, because the columns of $\bar{B}$ are independent and the virtual array is weighted ULA, the steering matrix $A_{E}$ and $A_{r}$ shown in Equaitons (19) and (23) are unambiguous.

Then according to the orthogonality principle of MUSIC algorithm [7,8], the real-valued steering matrix $A_{r}$ is orthogonal to the noise subspace $E_{n}$, that is

(29) $$\begin{array}{c} \left|\right|E_{n}^{H}A_{r}{\left|\right|}_{F}=0. \end{array}$$

Based on the structure of real-valued steering matrix in Equation (23), a new real-valued NC-MUSIC spectrum function is defined by

(30) $$\begin{array}{c} f\left(\theta_{l}\right)=\frac{1}{\det\{A_{r}^{H}\left({\bar{\theta }}_{l}\right)E_{n}E_{n}^{H}A_{r}\left({\bar{\theta }}_{l}\right)\}}. \end{array}$$

Using the discretized spatial sampling grid ${\left\{{\bar{\theta }}_{l}\right\}}_{l=1}^{K}\left(K\gg P\right)$, the spectrum function in Equation (30) can be used to formulate the weight vector $\gamma =[\gamma_{1},\gamma_{2},\cdots ,\gamma_{K}]$, which is shown as

(31) $$\begin{array}{c} \gamma_{l}=\det\left\{A_{r}^{H}\left({\bar{\theta }}_{l}\right)E_{n}E_{n}^{H}A_{r}\left({\bar{\theta }}_{l}\right)\right\},\;\;l=1,2,\cdots ,K. \end{array}$$

Then the weight matrix is designed as

(32) $$\begin{array}{c} W=\mathrm{diag}\{\gamma /\max(\gamma )\}. \end{array}$$

Due to the property of NC-MUSIC spectrum function, the elements $W\left(i,i\right)=\gamma_{i}//\max\left(\gamma \right)\to 0\left(i=1,2,\cdots ,P\right)$ corresponding to the possible sources are much smaller than the residual elements $W\left(i,i\right)=\gamma_{i}/\max\left(\gamma \right)$(i=1,2,⋯,L−P), which achieves the same idea of the reweighting the $l_{1}$-norm penalty in [30]. Finally, the real-valued reweighted nuclear norm minimization framework can be formulated as

(33) $$\begin{array}{c} \min_{S_{\bar{\theta }}}\sum_{l=1}^{K}\gamma_{l}\left|\right|S_{\bar{\theta }l}{\left|\right|}_{*}\\ s.t.\;||Z_{rs}-A_{\bar{\theta }}S_{\bar{\theta }}{\left|\right|}_{F}\leq \beta . \end{array}$$

Equation (33) can be solved by semidefinite programming (SDP) [29]. Due to the sparse rank in each block, the DOA can be achieved by plotting the spatial spectrum of $\tilde{S}=\left[|\right|S_{\bar{\theta }l}{\left|\right|}_{*},\cdots ,||S_{\bar{\theta }K}{\left|\right|}_{*}]$.

## 4. Related Remarks

**Remark** **1.** In the sparse recovery framework, the regularization parameter β plays an important role in the final DOA estimation performance, which sets the amount of error. The choice of regularization parameter β depends the probability distribution of the noise matrix $N_{rs}$. Based on [14], the noise matrix $N_{rs}$ has approximately a $\chi^{2}$ distribution with 2QP degrees of freedom upon normalization by a variance $\sigma^{2}/2$, where $\sigma^{2}$ is the noise variance. Thus, the regularization parameter β can be set as the upper value of $∥N_{rs}{∥}_{F}^{2}$ with a high probability 1−ξ, where ξ=0.01 is generally enough.

**Remark** **2.** In the proposed method, the noise variance is reduced by one half compared with the complex-valued sparse recovery based methods. In addition, the proposed method uses both the noncircularity of signals and reweighted matrix to enhance the sparsity of the solution. Thus, the proposed method is expected to have better angle estimation performance than conventional sparse recovery based algorithms.

**Remark** **3.** According to Equaiton (8), there are Q degrees of freedom (DOF) in the colocated MIMO radar considered in this paper. Thus, the maximum number of identifiable sources is Q−1 in the conventional sparse representation based algorithms. However, the proposed method uses the noncircularity of signals to enlarge the aperture of array in Equation (9), then the DOF is up to 2Q−1. Thus, the maximum number of identifiable sources of the proposed method is 2(Q−1), which indicates that the proposed method can identify more sources than conventional SR based algorithms and handle the case of underdetermined DOA estimation.

**Remark** **4.** According to Ref. [31], the SDP can be solved in $O(n_{1}^{2}n_{2}^{2.5})$ flops, where $n_{1}$ and $n_{2}\times n_{2}$ are the variable size and dimension of the positive semidefinite matrix in the semidefinite constraint of an SDP, respectively. In Equation (27), the variable size and dimension of the positive semidefinite matrix are 2PK and 2+P, respectively. Thus, the computational complexity of NNM algorithm is $O(4K^{2}P^{2}{\left(2+P\right)}^{2.5})$ flops. Due to the real-valued processing of the proposed method, its computational complexity is $O(K^{2}P^{2}{\left(2+P\right)}^{2.5})$ flops. In addtion, the computational complexity of $l_{1}$-SVD algorithm is $O(KP^{3})$ flops, therefore the computational complexity of the proposed method is higher than $l_{1}$-SVD algorithm.

## 5. Simulation Results

In order to verify the performance of the proposed method, simulation results are presented in this section. The proposed method is compared with the $l_{1}$-SVD algorithm [11], the NNM algorithm [27] and the the Cramér-Rao Bound (CRB) [27]. Unless otherwise stated in the following simulation results, a colocated MIMO radar equipped with M=4 transmit antennas and N=6 receive antennas is considered, and the transmit and receive arrays are half-wavelength spaced ULAs. on the transmit side, strictly noncircular waveforms, such as BPSK modulated signals, are emitted by M=4 transmit antennas. It is assumed that the number of sources is known or estimated by MDL criterion, and there are P=3 uncorrelated sources in the far field with the DOAs of $\theta_{1}=-10^{\circ }$, $\theta_{2}=0^{\circ }$ and $\theta_{3}=8^{\circ }$. The definition of signal-to-noise ratio (SNR) is given as 10log$\left(\sigma_{s}^{2}/\sigma_{n}^{2}\right)$, where $\sigma_{s}^{2}$ and $\sigma_{n}^{2}$ are the signal and noise power, respectively. The root-mean-square-error (RMSE) used to evaluate the DOA estimation is defined as

(34) $$\begin{array}{c} \mathrm{RMSE}=\sqrt{\frac{1}{\zeta P}\sum_{i=1}^{\zeta }\sum_{p=1}^{P}{\left({\hat{\theta }}_{i,p}-\theta_{p}\right)}^{2}} \end{array}$$

where ${\hat{\theta }}_{i,p}$ is the estimation of $\theta_{p}$ from the *i*th trial, and ζ is the total number of Monte Carlo trials. The discretized spatial sampling grid is set as $0.01^{\circ }$ for all the methods.

**Figure 2:**
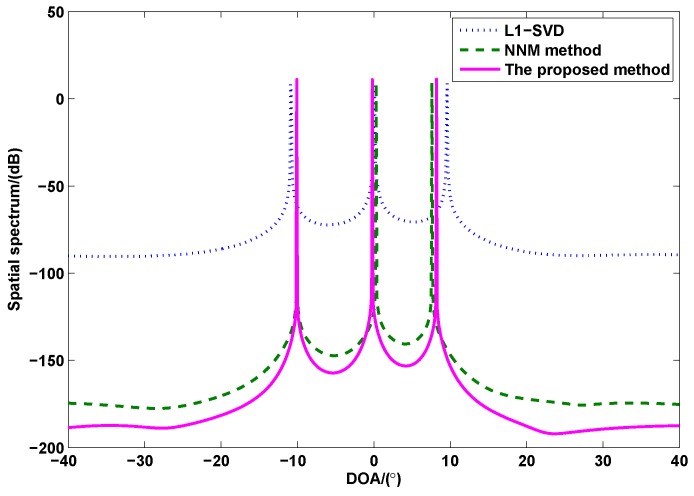
The spatial spectrum of all methods (*M* = 4, *N* = 6, SNR = 0 dB, *L* = 100).

Figure 2 shows the spatial spectrum of all methods, where the SNR is 0 dB, and the number of snapshots is L=100. From Figure 2, it can be seen that the proposed method and NNM method have sharper peaks and lower sidelobes than the $l_{1}$-SVD method, which indicates that they have better performance than $l_{1}$-SVD method. In addition, it is also noticed that the proposed method may have better performance than NNM method due to the ability of noise suppression.

**Figure 3:**
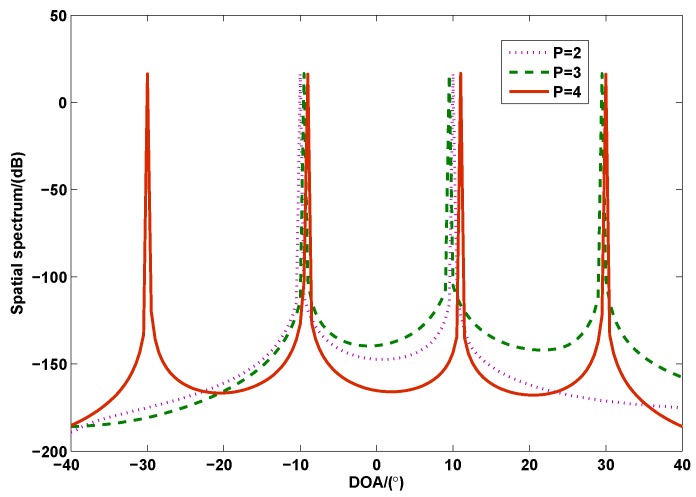
The spatial spectrum of the proposed method with different number of sources (*M* = *N* = 2, SNR = 10 dB, *L* = 100).

Figure 3 shows the spatial spectrum of the proposed method with different number of sources, where M=N=2, the SNR is 10 dB and the number of snapshots is L=100. There are three cases considered in this simulation. In case 1, two sources with DOAs of $-10^{\circ }$ and $10^{\circ }$ are considered. In case 2, three sources with DOAs of $-10^{\circ }$, $10^{\circ }$ and $30^{\circ }$ are considered. In the last case, there are four sources and the DOAs are $-10^{\circ }$ , $10^{\circ }$, $30^{\circ }$ and $-30^{\circ }$. According to the theoretical analysis, the maximum number of identifiable sources of the proposed method is 2(Q−1)=4 when M=N=2. As seen in Figure 3, the proposed method can correctly estimate the DOAs with P=4 sources when the DOF of the colocated MIMO radar is Q=3, which verifies that the proposed method can handle the case of underdetermined DOA estimation.

**Figure 4:**
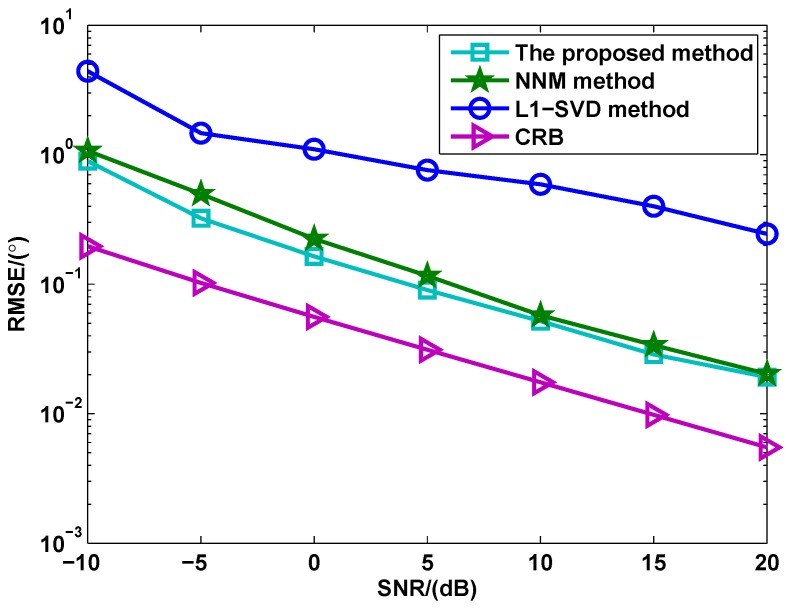
RMSE versus SNR for different methods (M=4,N=6,L=100).

Figure 4 shows the RMSE versus SNR for different methods, where ζ=100 and the number of snapshots is L=100. From Figure 4, both the proposed method and NNM method provide better DOA estimation performance than $l_{1}$-SVD method. The main reason is that they use the noncircularity of signals to enlarge the array aperture. On the other hand, it is also shown that the proposed method has superior performance than NNM method, especially in low SNR region. This is because that the proposed method uses the real structure to suppress the noise. It should be highlighted that the implementation of the proposed method is referred to as real-valued processing, which is different with the complex-valued implementation of NNM method.

**Figure 5:**
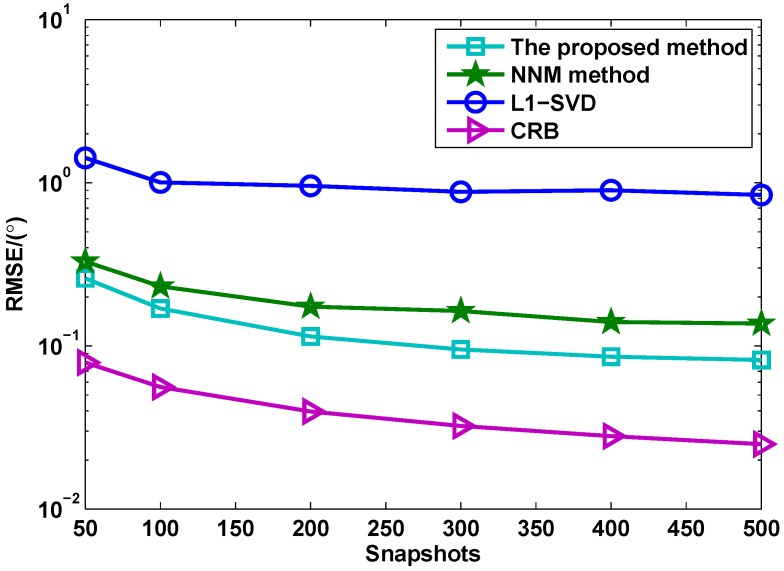
RMSE versus snapshots for different methods (*M* = 4, *N* = 6, SNR = 0 dB).

Figure 5 shows the RMSE versus the number of snapshots for different methods, where ζ=100 and SNR = 0 dB. It is shown that the DOA estimation performance of all methods can become better with the increasing number of the snapshots. The proposed method has the best performance compared with NNM and $l_{1}$-SVD methods due to its advantages.

**Figure 6:**
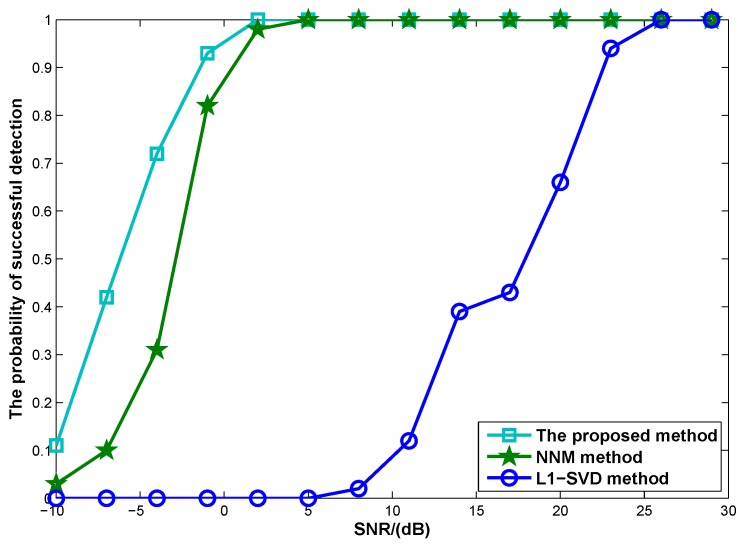
The probability of successful detection versus SNR (*M* = 4, *N* = 6, *L* = 100).

Figure 6 shows the probability of successful detection versus SNR, where the number of snapshots is L=100 and the number of Monte Carlo trials is set as 100. In this simulation, all sources can be considered as successful detection if the DOA estimation error satisfies $max_{i=1,2,3}|{\hat{\theta }}_{i}-\theta_{i}|\leq 0.5^{\circ }$, where ${\hat{\theta }}_{i}$ is the estimation of $\theta_{i}$. It can be seen from Figure 6 that all methods can achieve 100% detection performance when the SNR is high enough. The SNR threshold is defined as a point at which the probability of successful detection starts dropping. According to Figure 6, it is clearly shown that the proposed method has lowest SNR threshold compared with NNM and $l_{1}$-SVD methods, i.e., the proposed method provides better resolution than both of them.

**Figure 7:**
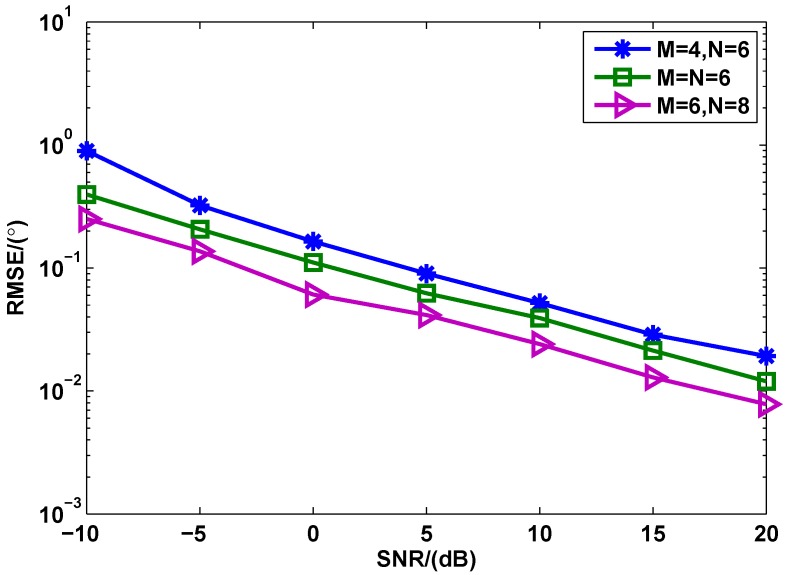
RMSE of the proposed method with different number of transmit/receive elements (L=100).

Figure 7 shows the RMSE of the proposed method with different number of tranmit/receive elements. The RMSE of the proposed method becomes smaller at the same SNR when the number of transmit or/and receive elements increases. The key reason is that the more transmit or/and receive elements the MIMO radar has, the more spatial gain can be obtained.

## 6. Conclusions

In this paper, we have proposed a unitary nuclear norm minimization algorithm for DOA estimation with noncircular source in MIMO radar. The proposed method uses the noncircular properties of signals to achieve the extended data, and the unitary transformation is utilized to obtain the real-valued data and overcomplete dictionary. The DOA is estimated by formulating the real-valued reweighted nuclear norm minimization framework based on the block sparse model.The computational complexity of the proposed method has been analyzed, and it has been shown that the computational complexity of the proposed method is higher than $l_{1}$-SVD algorithm. The simulation results have verified that the proposed method provides better DOA estimation performance and higher resolution than the existing sparse recovery based methods. It is also shown that the proposed method can handle the case of underdetermined DOA estimation.
